# Evaluation and study of adverse reactions to imiglucerase based on the FAERS database

**DOI:** 10.1186/s13023-025-03934-7

**Published:** 2025-08-07

**Authors:** Pan Hong, Qingbo Zhou

**Affiliations:** 1 Department of Hematology, Shaoxing People’s Hospital, Shaoxing, Zhejiang China; 2 Department of Internal Medicine, Shaoxing Yuecheng People’s Hospital, Shaoxing, Zhejiang China

**Keywords:** Imiglucerase, Gaucher disease, Adverse drug reactions, FAERS database, Pharmacovigilance

## Abstract

**Objective:**

This study aims to evaluate the adverse drug reactions associated with imiglucerase in the treatment of Gaucher disease by analyzing data from the FDA Adverse Event Reporting System (FAERS) database.

**Methods:**

A comprehensive analysis was conducted on 166,800,135 adverse event reports from the FAERS database, covering the period from the first quarter of 2004 to the fourth quarter of 2023. The data were processed using R software and analyzed using multiple disproportionality methods, including Reporting Odds Ratio (ROR), Proportional Reporting Ratio (PRR), Bayesian Confidence Propagation Neural Network (BCPNN), and Multi-Item Gamma Poisson Shrinker (MGPS). These methodologies were applied to identify significant adverse reaction signals across various System Organ Classes (SOCs) and Preferred Terms (PTs).

**Results:**

The analysis revealed significant adverse reaction signals in multiple SOCs, including general disorders and administration site conditions, injury, poisoning and procedural complications, infections and infestations, and nervous system disorders. Notably, general disorders and injury-related conditions had the highest number of reports. At the PT level, the term "Gaucher disease" yielded the highest statistical signal. This was identified as a critical reporting artifact, likely representing perceived treatment failure or disease progression, rather than a true adverse reaction. After accounting for this artifact, other significant adverse event signals included increased chitotriosidase, elevated acid phosphatase, and bone infarction, with musculoskeletal and connective tissue disorders being a key area of concern. A comparative analysis against other Gaucher therapies suggests this strong skeletal signal likely reflects confounding by indication rather than a drug-specific risk.

**Conclusion:**

The findings underscore the importance of ongoing pharmacovigilance to monitor the safety of imiglucerase, especially among vulnerable populations such as pregnant women, long-term users, and those with comorbid hepatobiliary or skeletal conditions.

**Supplementary Information:**

The online version contains supplementary material available at 10.1186/s13023-025-03934-7.

## Introduction

Gaucher disease is a rare inherited metabolic disorder caused by a deficiency of the enzyme glucocerebrosidase. According to the World Health Organization, approximately 6,000 individuals worldwide are affected by Gaucher disease, with a lifetime prevalence estimated to be as high as 1 in 100,000 individuals [1]. In the United States, Gaucher disease is relatively rare, with recent estimates indicating that there are approximately 100–200 new diagnoses annually [2, 3]. Despite the availability of enzyme replacement therapy (ERT) and other supportive treatments, a subset of patients exhibit suboptimal responses to these therapies, and the treatment costs remain prohibitively high [4]. Imiglucerase, the primary therapeutic agent for Gaucher disease, works by supplementing the deficient enzyme in patients, effectively reducing symptoms such as splenomegaly, hepatomegaly, bone pathology, and hematologic abnormalities [5].

The etiology of Gaucher disease is multifaceted, involving genetic, environmental, enzymatic deficiency, and metabolic dysregulation factors [1]. This complexity necessitates diverse treatment strategies and personalized therapeutic approaches [4]. In this context, effective and safe pharmacological treatments are crucial [2]. Imiglucerase was approved by the FDA in 1994 for the treatment of Gaucher disease. However, the FDA Adverse Event Reporting System (FAERS) database has only provided publicly available data from the first quarter of 2004 onward, making it the earliest available starting point for this analysis [6]. Further investigations have also demonstrated that imiglucerase remains highly effective in improving the quality of life in patients, including those who initiated treatment later in the disease progression [7].

However, like all pharmacological treatments, imiglucerase is associated with the potential risk of adverse drug reactions (ADRs) [8]. The FDA Adverse Event Reporting System (FAERS) provides a platform for collecting and analyzing adverse events (ADEs) associated with drug use [9]. These data are critical resources for evaluating the safety and efficacy of medications [10]. This study aims to mine data related to imiglucerase from the FAERS database, utilizing various signal detection methodologies to assess the data from multiple perspectives, thereby providing more comprehensive and reliable results [11]. The findings of this research will further elucidate the safety profile of imiglucerase and its clinical efficacy in treating Gaucher disease, offering valuable insights for clinicians and patients [12]. To better contextualize these signals and address the critical challenge of confounding by indication, this study also compares the adverse event profile of imiglucerase against other approved therapies for Gaucher disease.

## Methodology

### Data source, processing, and signal detection

This study utilized data from the FDA Adverse Event Reporting System (FAERS) for the period spanning from the first quarter (Q1) of 2004 to the fourth quarter (Q4) of 2023. The selection of 2004 as the starting year was dictated by the public availability of FAERS's structured quarterly data files, which ensures that the analysis is based on comprehensive and consistent data.

**Fig. 1:**
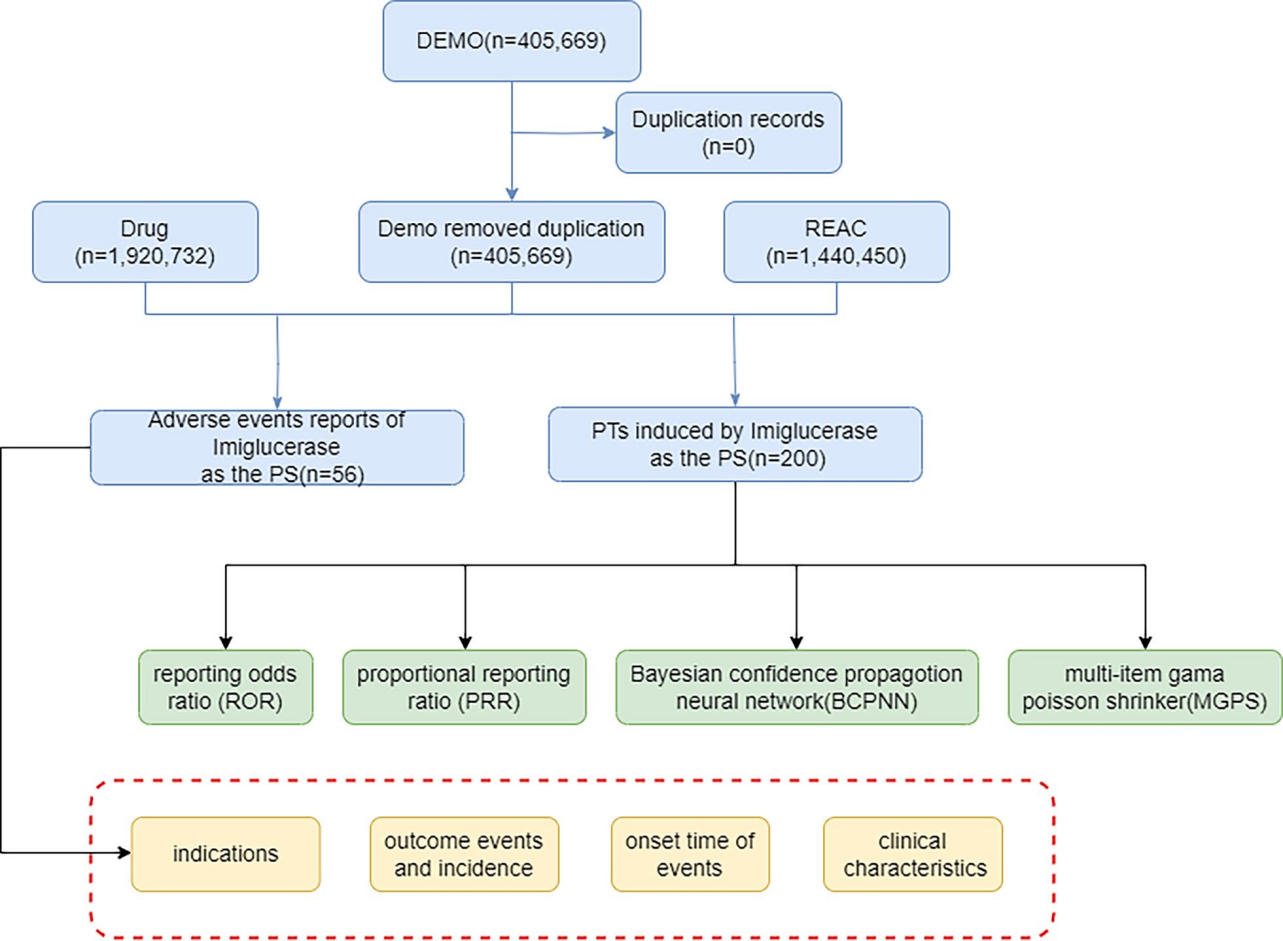
Flowchart depicting the selection process of imiglucerase-related adverse events (AEs) from the FAERS database

Data processing was systematically conducted using R software. Specifically, the FAERS R package was employed to facilitate complex tasks. To ensure comprehensive retrieval of all relevant case reports, our drug name query included the generic name ('Imiglucerase'), the primary brand name ('Cerezyme'), and its Chinese translation ('伊米苷酶'), leveraging the package's multi-language capabilities. The package then handled subsequent data integration, standardization, and deduplication. A comprehensive list of all drug name query terms used for this retrieval is provided as a supplementary file (Supplementary Table 1). Duplicate reports were meticulously removed by retaining only the most recent report for each unique case. Furthermore, relationships between datasets were established, and outliers, particularly within age and weight data, were corrected to maintain data integrity and accuracy (Fig. 1).

To identify potential safety signals, this study employed a range of established disproportionality analysis methods, including the Reporting Odds Ratio (ROR), the Proportional Reporting Ratio (PRR), the Bayesian Confidence Propagation Neural Network (BCPNN), and the Multi-item Gamma Poisson Shrinker (MGPS). The combined use of these algorithms provides a more comprehensive and robust signal detection framework. A 2 × 2 contingency table (Table 1) was constructed to support these analyses, outlining the relationship between the drug of interest and all other drugs concerning the reported adverse events. The specific formulas and thresholds applied to refine this signal detection process are detailed in Table 2.

**Table 1:** The 2 × 2 contingency table for disproportionality analysis

	Target drug	Other drugs	Total
Adverse events of interest	a	b	a + b
Other adverse events	c	d	c + d
Total	a + c	b + d	a + b + c + d

The variables are defined as follows: **a**, the number of reports for the target drug and the adverse event of interest; **b**, the number of reports for other drugs and the adverse event of interest; **c**, the number of reports for the target drug and all other adverse events; **d**, the number of reports for all other drugs and all other adverse events

**Table 2:** Signal detection algorithms, formulas, and established thresholds used in the analysis

Method	Formula	Threshold
PRR	PRR = (a/(a + b))/(c/(c + d))	PRR ≥ 2 and lower CI > 1
ROR	ROR = (a/c)/(b/d)	a ≥ 3 and 95% CI > 1
BCPNN	IC = log2((p(x,y))/(p(x)p(y)))	IC025 > 0
EBGM	EBGM = exp((a + c)/(b + d))	EBGM05 > 2

PRR, Proportional Reporting Ratio; ROR, Reporting Odds Ratio; BCPNN, Bayesian Confidence Propagation Neural Network; IC, Information Component; IC025, the lower bound of the 95% confidence interval for IC; EBGM, Empirical Bayes Geometric Mean; EBGM05, the lower bound of the 95% confidence interval for EBGM; CI, Confidence Interval. The variables a, b, c, and d are defined in Table 1

*The EBGM formula shown is a simplified representation; the actual calculation is based on a Gamma-Poisson model.*

#### Rationale for signal detection thresholds

The selection of thresholds for disproportionality analysis is a critical step to balance sensitivity (the ability to detect true signals) and specificity (the ability to avoid false positives). The criteria used in this study (Table 2) are aligned with established guidelines from regulatory authorities and best practices in pharmacovigilance research to ensure the statistical robustness of our findings.

- ROR and PRR: For the Reporting Odds Ratio (ROR), a signal was considered noteworthy if it met two criteria: at least three case reports (a ≥ 3) and the lower bound of the 95% confidence interval (95% CI) > 1. The a ≥ 3 criterion ensures that the signal is not based on anecdotal cases and provides statistical stability. Similarly for the Proportional Reporting Ratio (PRR), the threshold of PRR ≥ 2 serves as an initial flag, but the core requirement is that its lower CI > 1. For both methods, a lower CI bound greater than 1 indicates that the observed disproportionality is statistically significant and unlikely to be due to random chance [13, 14].
- BCPNN: The Bayesian Confidence Propagation Neural Network (BCPNN) calculates an Information Component (IC). We adopted the standard criterion established by the World Health Organization's Uppsala Monitoring Centre (WHO-UMC), which defines a signal when the lower end of the IC's 95% credibility interval (IC025) is greater than 0. A positive IC025 value suggests with high confidence that the number of observed reports for a drug-event combination is statistically greater than the number expected based on the overall reporting frequency in the database [15].
- MGPS: The Multi-item Gamma Poisson Shrinker (MGPS) is an algorithm developed and used by the U.S. Food and Drug Administration (FDA) for signal detection. We employed the FDA's conservative threshold, which requires the lower bound of the 95% CI of the Empirical Bayes Geometric Mean (EBGM), denoted as EBGM05, to be greater than 2. This is a particularly stringent criterion. The EBGM is a"shrunken"estimate that moderates ratios from small counts toward the background rate, making it more stable. Requiring the EBGM05 > 2 provides high confidence (95%) that the true reporting ratio is at least double the background rate, thereby enhancing signal reliability and minimizing false-positive associations [16–18].

By concurrently applying these methodologically diverse and well-validated thresholds, we aimed to generate a high-quality list of signals for imiglucerase, ensuring our analysis was both robust and conservative.

#### Handling of missing data and outliers

The analysis of real-world data from spontaneous reporting systems like FAERS necessitates a clear strategy for handling inevitable missing data and potential data entry errors. Our approach relied on the standardized procedures embedded within the FAERS R package, a tool specifically designed for FAERS data analysis.

Missing Data:

Spontaneous adverse event reports are often incomplete. In our analysis, we did not perform data imputation for missing categorical variables (e.g., sex, reporter country). Instead, to avoid the selection bias that would arise from excluding cases with incomplete information, we retained all 3,810 unique case reports in our analytical cohort. When generating descriptive statistics (Table 3), the AER_tab1 function from the FAERS R package automatically handles missing values by tabulating them into a separate"unknown"or"not specified"category. This approach ensures transparency regarding data completeness and is a standard practice in pharmacovigilance studies.

**Table 3:** Demographic and clinical characteristics of adverse event reports for imiglucerase in the FAERS database (2004–2023)

Variable	Total
*Year*	
2004	110 (2.89)
2005	98 (2.57)
2006	120 (3.15)
2007	161 (4.23)
2008	167 (4.38)
2009	226 (5.93)
2010	206 (5.41)
2011	223 (5.85)
2012	145 (3.81)
2013	223 (5.85)
2014	320 (8.40)
2015	246 (6.46)
2016	207 (5.43)
2017	220 (5.77)
2018	160 (4.20)
2019	203 (5.33)
2020	156 (4.09)
2021	149(3.91)
2022	216 (5.67)
2023	254 (6.67)
*Sex*	
Female	1980 (51.97)
Male	1384 (36.33)
Unknown	446 (11.71)
*Age_yr*	33.00 (13.00,57.00)
*Wt*	57.60 (28.12,74.00)
*Reporter*	
Physician	1762 (46.25)
Consumer	1264 (33.18)
Other health-professional	523 (13.73)
Pharmacist	213 (5.59)
unknown	45 (1.18)
Registered Nurse	3 (0.08)
*Reported countries*	
Known source	2406 (63.15)
United States	1507 (62.64)^a^
*Other*	561 (23.32)^a^
Brazil	87 (3.62)^a^
Egypt	79 (3.28)^a^
United Kingdom	66 (2.74)^a^
France	56 (2.33)^a^
Japan	50 (2.08)^a^
Unknown source	1404 (36.85)
*Route*	
Intravenous	2488 (65.30)
Intravenous drip	875 (22.97)
Other	428 (11.23)
Transplacental	19 (0.50)
*Outcomes b*	
Other serious	1425 (38.80)
Hospitalization	1277 (34.77)
Death	744 (20.26)
Life threatening	98 (2.67)
Disability	91 (2.48)
Required intervention to Prevent Permanent Impairment/Damage	32 (0.87)
Congenital anomaly	6 (0.16)
*tto*	1117.00 (246.50,3118.50)

Data are presented as n (%) for categorical variables and median (Interquartile Range, IQR) for continuous variables

^a^Percentages for specific countries are calculated from the subtotal of reports with a known country of origin (N = 2406)

^b^Percentages for Outcomes are calculated from the subtotal of reports with available outcome data (N = 3673), excluding 137 reports where this information was missing

Outliers and Data Integrity:

The initial data processing and cleaning were managed by the core functions of the FAERS R package, primarily the screen() function. Pharmacovigilance analysis packages like FAERS R are designed to include internal data integrity checks. These automated pipelines are crucial for handling the known issues in raw FAERS data, which can include clinically implausible values or data entry errors in continuous variables like age and weight. By using this specialized package, we ensured that our analysis was based on a dataset that had undergone a standardized and replicable cleaning process, which is essential for maintaining the integrity and accuracy of the descriptive and inferential statistics.

### Signal filtering and classification

For the initial signal screening, a case frequency threshold was established, including only Preferred Terms (PTs) with three or more reports. Subsequently, all qualifying signals were coded and classified according to the Medical Dictionary for Regulatory Activities (MedDRA) hierarchy. This process involved mapping the signals to their respective System Organ Classes (SOCs) to enable a systematic analysis of the organ systems involved.

### Comparative analysis to address confounding by indication

To evaluate and address potential"confounding by indication"—the challenge of distinguishing adverse events caused by a drug from those caused by the underlying disease—we introduced a comparative signal analysis. This analysis included three other FDA-approved therapies for Gaucher disease: two additional enzyme replacement therapies (ERTs), Velaglucerase alfa and Taliglucerase alfa, and one substrate reduction therapy (SRT), Eliglustat. The same data extraction and signal detection methodologies used for imiglucerase were applied to these comparator drugs to assess differences in signal strength for key adverse events, particularly those related to the skeletal system.

To ensure full transparency and the reproducibility of our findings, all data processing and statistical analyses were conducted using the R programming language (Version 4.3.1) with the faersR package (Version 0.0.0.9008). The complete R script detailing the primary analysis of imiglucerase adverse event reports presented in this study is provided as Supplementary File S1. Furthermore, the scripts used for the comparative analyses of velaglucerase alfa, taliglucerase alfa, and eliglustat are available as Supplementary Files S2–S4.

## Results

### Data overview and trend analysis

**Fig. 2:**
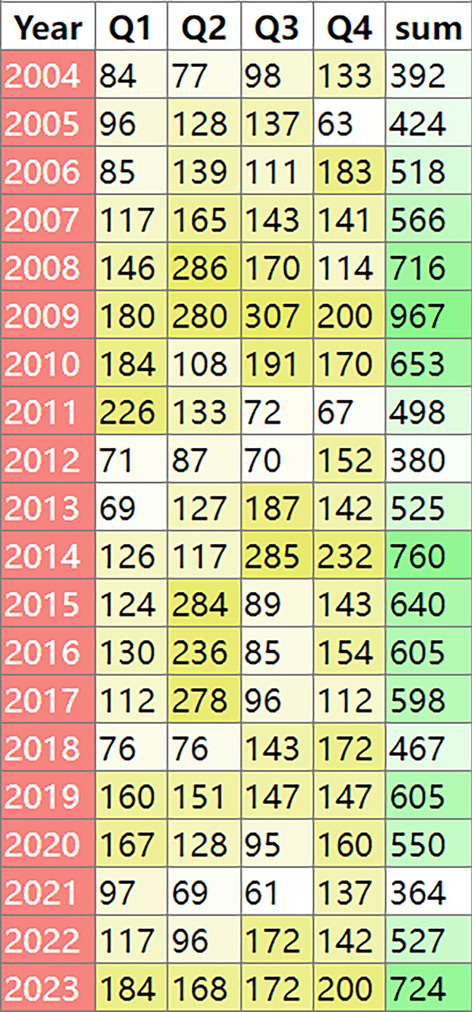
Annual and quarterly distribution of adverse event reports (AERs) Related to Imiglucerase from FAERS Database

This study analyzed 166,800,135 adverse event reports from the FAERS database, spanning from the first quarter of 2004 to the fourth quarter of 2023. The data indicate that the annual number of reports peaked in 2009 with 967 reports and declined to a low of 364 reports in 2021. Overall, the number of reports has shown a declining trend since 2018, but there was a resurgence in 2023, with 724 reports recorded. The quarterly distribution revealed significant fluctuations between years, with a peak in the second quarter of 2008 (286 reports), whereas the report numbers were relatively even across the quarters in 2023. These data provide insights into the temporal changes of adverse reactions associated with imiglucerase and their impact over different time periods (Fig. 2).

### Basic characteristics of adverse drug reactions (ADRs) associated with imiglucerase

During the study period from the first quarter of 2004 to the fourth quarter of 2023, after a meticulous deduplication and filtering process as detailed in Fig. 1, a final dataset of 3,810 unique case reports identifying imiglucerase as the primary suspected drug was established for analysis. The basic demographic and clinical characteristics of these reports are summarized in Table 3. Among these reports involving imiglucerase, female patients were significantly more represented than male patients, accounting for 51.97% and 36.33%, respectively. The average age of the reported patients was 33 years.

The majority of the reports were submitted by internists (46.25%) and consumers (33.18%). At least half of the reports originated from countries with unknown sources, while among those with known origins, the United States accounted for the most, representing 39.55% of the reports. Regarding the route of administration, the vast majority of cases involved intravenous injection (65.30%).

In terms of clinical outcomes, the most common adverse events were those resulting in hospitalization or prolonged hospital stay, which accounted for 34.77% of the reports, followed by death events at 20.26%. Other notable adverse events included life-threatening conditions (2.67%) and disability (2.48%). The median duration of drug use was 1,117 days (interquartile range: 246.50–3118.50 days). These data provide an overview of the fundamental characteristics of imiglucerase use in clinical practice (Table 3).

### Analysis of imiglucerase signal mining at the SOC level

Through signal detection, the study analyzed the distribution of adverse reactions associated with imiglucerase across different System Organ Classifications (SOCs). The results revealed that the SOCs with the highest number of reports were:

1. General disorders and administration site conditions (n = 1699)
2. Injury, poisoning, and procedural complications (n = 1245)
3. Infections and infestations (n = 1155)
4. Nervous system disorders (n = 1015)

In terms of signal strength, imiglucerase was highly associated with adverse events in the following SOC categories:

1. Pregnancy, puerperium, and perinatal conditions (n = 161): ROR 3.1 (95% CI: 2.65, 3.62), PRR 3.07 (95% CI: 2.62, 3.59), chisq 225.32, IC 1.62 (1.39), EBGM 3.07 (2.69).
2. Congenital, familial, and genetic disorders (n = 90): ROR 2.41 (95% CI: 1.96, 2.97), PRR 2.4 (95% CI: 1.93, 2.98), chisq 73.89, IC 1.26 (0.97), EBGM 2.4 (2.02).
3. Hepatobiliary disorders (n = 225): ROR 2.08 (95% CI: 1.82, 2.37), PRR 2.06 (95% CI: 1.8, 2.36), chisq 123.42, IC 1.04 (0.85), EBGM 2.06 (1.84).

These results highlight the distribution and signal strength of adverse reactions associated with imiglucerase across different SOC categories, indicating potential safety concerns in the drug's practical use (Table 4).

**Table 4:** Signal detection analysis for imiglucerase at the System Organ Class (SOC) level

SOC	Case Reports	ROR (95% CI)	PRR (95% CI)	Chisq	IC (IC025)	EBGM (EBGM05)
Pregnancy, puerperium and perinatal conditions	161	3.1 (2.65, 3.62)	3.07 (2.62, 3.59)	225.32	1.62 (1.39)	3.07 (2.69)
Congenital, familial and genetic disorders	90	2.41 (1.96, 2.97)	2.4 (1.93, 2.98)	73.89	1.26 (0.97)	2.4 (2.02)
Hepatobiliary disorders	225	2.08 (1.82, 2.37)	2.06 (1.8, 2.36)	123.42	1.04 (0.85)	2.06 (1.84)
Infections and infestations	1155	1.94 (1.83, 2.06)	1.85 (1.74, 1.96)	473.15	0.88 (0.8)	1.85 (1.75)
Blood and lymphatic system disorders	327	1.63 (1.46, 1.82)	1.61 (1.46, 1.78)	76.66	0.69 (0.53)	1.61 (1.47)
Respiratory, thoracic and mediastinal disorders	895	1.61 (1.51, 1.73)	1.56 (1.47, 1.65)	191.48	0.65 (0.55)	1.56 (1.48)
Musculoskeletal and connective Tissue disorders	910	1.47 (1.37, 1.57)	1.43 (1.35, 1.52)	125.27	0.52 (0.42)	1.43 (1.35)
Vascular disorders	350	1.35 (1.21, 1.5)	1.34 (1.21, 1.48)	30.33	0.42 (0.27)	1.34 (1.22)
Cardiac disorders	416	1.29 (1.17, 1.42)	1.28 (1.16, 1.41)	26.23	0.36 (0.22)	1.28 (1.18)
Investigations	875	1.19 (1.11, 1.27)	1.17 (1.1, 1.24)	23.44	0.23 (0.13)	1.17 (1.11)
Injury, poisoning and procedural complications	1245	1.17 (1.11, 1.24)	1.15 (1.08, 1.22)	28.43	0.21 (0.12)	1.15 (1.1)
Nervous system disorders	1015	0.99 (0.93, 1.05)	0.99 (0.93, 1.05)	0.12	− 0.01 (− 0.11)	0.99 (0.94)
Neoplasms benign, malignant and Unspecified (incl cysts and polyps)	317	0.98 (0.87, 1.09)	0.98 (0.87, 1.1)	0.2	− 0.04 (− 0.2)	0.98 (0.89)
Ear and labyrinth disorders	47	0.91 (0.68, 1.21)	0.91 (0.68, 1.22)	0.45	− 0.14 (− 0.55)	0.91 (0.71)
General disorders and Administration site conditions	1699	0.78 (0.74, 0.82)	0.82 (0.79, 0.85)	86.85	− 0.29 (− 0.37)	0.82 (0.78)
Metabolism and nutrition disorders	203	0.78 (0.68, 0.9)	0.79 (0.69, 0.91)	12.11	− 0.35 (− 0.55)	0.79 (0.7)
Immune system disorders	84	0.63 (0.51, 0.78)	0.64 (0.52, 0.79)	17.74	− 0.65 (− 0.96)	0.64 (0.53)
Gastrointestinal disorders	667	0.63 (0.58, 0.68)	0.65 (0.6, 0.7)	138.07	− 0.62 (− 0.73)	0.65 (0.61)
Eye disorders	135	0.56 (0.47, 0.66)	0.56 (0.47, 0.67)	46.22	− 0.82 (− 1.07)	0.56 (0.49)
Renal and urinary disorders	125	0.56 (0.47, 0.66)	0.56 (0.47, 0.67)	43.96	− 0.84 (− 1.09)	0.56 (0.48)
Endocrine disorders	13	0.43 (0.25, 0.74)	0.43 (0.25, 0.74)	9.74	− 1.21 (− 1.97)	0.43 (0.27)
Skin and subcutaneous tissue disorders	279	0.42 (0.37, 0.47)	0.44 (0.39, 0.49)	216.43	− 1.2 (− 1.37)	0.44 (0.39)
Reproductive system and breast Disorders	39	0.39 (0.28, 0.53)	0.39 (0.29, 0.53)	37.94	− 1.37 (− 1.81)	0.39 (0.3)
Psychiatric disorders	207	0.29 (0.25, 0.33)	0.3 (0.26, 0.34)	363.8	− 1.75 (− 1.94)	0.3 (0.27)

SOCs are sorted by the Reporting Odds Ratio (ROR) in descending order. Signals are considered positive if the lower bound of the 95% confidence interval for ROR is > 1. Abbreviations: SOC, System Organ Class; CI, Confidence Interval; ROR, Reporting Odds Ratio; PRR, Proportional Reporting Ratio; $\chi^2$, Chi-square; IC, Information Component; IC025, lower bound of the 95% CI for IC; EBGM, Empirical Bayes Geometric Mean; EBGM05, lower bound of the 95% CI for EBGM

### Analysis of imiglucerase at the PT level

At the Preferred Term (PT) level, the PT “Gaucher disease” yielded the strongest statistical signal, with an ROR of 6352.95 (95% CI: 4274.52, 9441.98). This signal is a well-documented reporting artifact inherent to pharmacovigilance analyses of therapies for specific diseases. It does not represent a drug-induced adverse event but rather reflects reports of"drug ineffectiveness”,"condition aggravated”, or disease progression, which are often coded in MedDRA under the indication's own PT.

Recognizing this as a case of indication-related reporting bias, this signal was methodologically excluded from the analysis of true adverse drug reactions.] After its exclusion, other clinically relevant signals with high strength were identified, including:

- Increased chitotriosidase: ROR 5077.83 (95% CI: 3014.21, 8554.28)
- Increased acid phosphatase: ROR 910.62 (95% CI: 376.98, 2199.65)
- Bone infarction: ROR 316.62 (95% CI: 217.03, 461.93)

In total, the study identified 101 PTs with high signal strength. These results suggest that multiple potential safety risks may be associated with the clinical use of imiglucerase (Table 5).

**Table 5:** Top 30 adverse event signals for imiglucerase at the Preferred Term (PT) level, ranked by Reporting Odds Ratio (ROR)

SOC	PT	Case reports	ROR (95% CI)	PRR (95% CI)	Chisq	IC (IC025)	EBGM (EBGM05)
Investigations	Chitotriosidase increased	31	5077.83 (3014.21, 8554.28)	5064.12 (2983.15, 8596.72)	71,580.25	11.17 (10.55)	2310.49 (1493.41)
Investigations	Blood acid phosphatase increased	6	910.62 (376.98, 2199.65)	910.14 (376.76, 2198.65)	4487.3	9.55 (8.38)	749.7 (358.43)
Investigations	Angiotensin converting enzyme increased	5	147.54 (60.48, 359.93)	147.48 (61.05, 356.27)	703.01	7.16 (5.98)	142.56 (67.6)
Investigations	Immunoglobulins increased	3	33.1 (10.63, 103.11)	33.1 (10.62, 103.17)	92.66	5.04 (3.62)	32.85 (12.7)
Injury, poisoning and procedural complications	Surgical procedure repeated	10	65.1 (34.85, 121.6)	65.04 (34.74, 121.78)	621.08	6 (5.14)	64.08 (37.99)
Injury, poisoning and procedural complications	Paternal exposure timing unspecified	3	53.11 (17, 165.85)	53.09 (17.03, 165.47)	151.44	5.71 (4.29)	52.45 (20.23)
Injury, poisoning and procedural complications	Respiratory fume inhalation disorder	3	49.79 (15.95, 155.42)	49.77 (15.97, 155.12)	141.72	5.62 (4.2)	49.21 (18.98)
Injury, poisoning and procedural complications	Drug exposure before pregnancy	6	27.15 (12.17, 60.61)	27.14 (12.15, 60.62)	150.1	4.75 (3.68)	26.97 (13.78)
Respiratory, thoracic and mediastinal disorders	Adenoidal hypertrophy	3	38.62 (12.39, 120.39)	38.61 (12.39, 120.34)	108.92	5.26 (3.84)	38.27 (14.78)
Respiratory, thoracic and mediastinal disorders	Apnoeic attack	4	33.26 (12.43, 88.97)	33.25 (12.48, 88.59)	124.14	5.04 (3.77)	33 (14.48)
Respiratory, thoracic and mediastinal disorders	Laryngospasm	11	19.78 (10.94, 35.78)	19.76 (10.98, 35.58)	195.06	4.3 (3.48)	19.68 (11.98)
Nervous system disorders	Pyramidal tract syndrome	4	47.74 (17.82, 127.92)	47.72 (17.91, 127.15)	180.94	5.56 (4.29)	47.2 (20.69)
Nervous system disorders	Myoclonic epilepsy	11	39.7 (21.92, 71.9)	39.66 (22.03, 71.4)	410.72	5.3 (4.48)	39.3 (23.91)
Nervous system disorders	Dementia with lewy bodies	4	24.59 (9.2, 65.73)	24.59 (9.23, 65.52)	89.99	4.61 (3.34)	24.45 (10.74)
Hepatobiliary disorders	Hepatosplenomegaly	22	41.44 (27.22, 63.09)	41.36 (27.4, 62.42)	858.22	5.36 (4.76)	40.97 (28.83)
Hepatobiliary disorders	Chronic hepatic failure	3	22.88 (7.36, 71.17)	22.88 (7.34, 71.31)	62.42	4.51 (3.09)	22.76 (8.81)
Hepatobiliary disorders	Portal hypertension	12	19.75 (11.2, 34.84)	19.73 (11.18, 34.83)	212.42	4.3 (3.51)	19.65 (12.22)
Blood and lymphatic system disorders	Bone marrow infiltration	7	95.97 (45.36, 203.03)	95.91 (45.54, 201.99)	642.91	6.55 (5.54)	93.81 (50.11)
Blood and lymphatic system disorders	Spleen disorder	12	34.31 (19.43, 60.57)	34.28 (19.42, 60.52)	384.57	5.09 (4.3)	34.01 (21.14)
Blood and lymphatic system disorders	Splenomegaly	59	25.02 (19.36, 32.34)	24.89 (19.29, 32.11)	1345.56	4.63 (4.26)	24.76 (19.97)
Neoplasms benign, malignant and unspecified (incl cysts and polyps)	Gammopathy	3	76.32 (24.36, 239.08)	76.3 (24.48, 237.81)	219	6.23 (4.8)	74.97 (28.84)
Neoplasms benign, malignant and unspecified (incl cysts and polyps)	Monoclonal gammopathy	3	23.52 (7.56, 73.15)	23.51 (7.54, 73.28)	64.3	4.55 (3.13)	23.39 (9.05)
Musculoskeletal and connective tissue disorders	Bone infarction	29	316.62 (217.03, 461.93)	315.83 (217.63, 458.34)	8471.24	8.2 (7.66)	294.04 (214.36)
Musculoskeletal and connective tissue disorders	Hip deformity	4	46.56 (17.38, 124.75)	46.55 (17.47, 124.03)	176.34	5.53 (4.25)	46.05 (20.19)
Infections and infestations	Epiglottitis	5	46.19 (19.13, 111.52)	46.17 (19.11, 111.53)	218.57	5.51 (4.35)	45.68 (21.85)
Infections and infestations	Dengue fever	12	30.4 (17.23, 53.66)	30.37 (17.2, 53.62)	338.48	4.91 (4.13)	30.17 (18.75)
Vascular disorders	Aortic dilatation	4	18.99 (7.11, 50.71)	18.98 (7.12, 50.57)	67.84	4.24 (2.97)	18.9 (8.31)
Pregnancy, puerperium and perinatal conditions	Twin pregnancy	4	21.54 (8.06, 57.54)	21.53 (8.08, 57.37)	77.92	4.42 (3.15)	21.43 (9.42)
Gastrointestinal disorders	Protein-losing gastroenteropathy	4	43.91 (16.4, 117.62)	43.9 (16.48, 116.97)	165.98	5.44 (4.17)	43.46 (19.06)
Congenital, familial and genetic disorders	Gaucher's disease	61	6352.95 (4274.52, 9441.98)	6319.19 (4269.9, 9352.01)	154,895.45	11.31 (10.86)	2540.67 (1823.71)

^*^The PT"Gaucher's disease"represents a known indication-related artifact and was excluded from the analysis of true adverse drug reactions, as detailed in the text. The full list of all 101 identified signals is available in Supplementary Table S1.*

SOC, System Organ Class; PT, Preferred Term; CI, Confidence Interval; ROR, Reporting Odds Ratio; PRR, Proportional Reporting Ratio; $\chi^2$, Chi-square; IC, Information Component; IC025, lower bound of the 95% CI for IC; EBGM, Empirical Bayes Geometric Mean; EBGM05, lower bound of the 95% CI for EBGM

### Analysis of PTs by SOC categories

Analyzing PTs by their corresponding SOC categories revealed that the musculoskeletal and connective tissue disorders category had the highest number of reports (n = 313). Within this category, common PTs included bone pain (n = 126) and osteonecrosis (n = 80). However, the most significant signal strengths were observed for bone infarction (ROR 316.62, 95% CI: 217.03, 461.93) and hip dysplasia (ROR 46.56, 95% CI: 17.38, 124.75).

These results indicate that imiglucerase may be associated with a higher risk of ADEs within specific SOC categories, particularly concerning musculoskeletal and connective tissue disorders during the treatment of Gaucher disease (Supplementary Table 2).

### Stability analysis of FAERS database annual reports

**Fig. 3:**
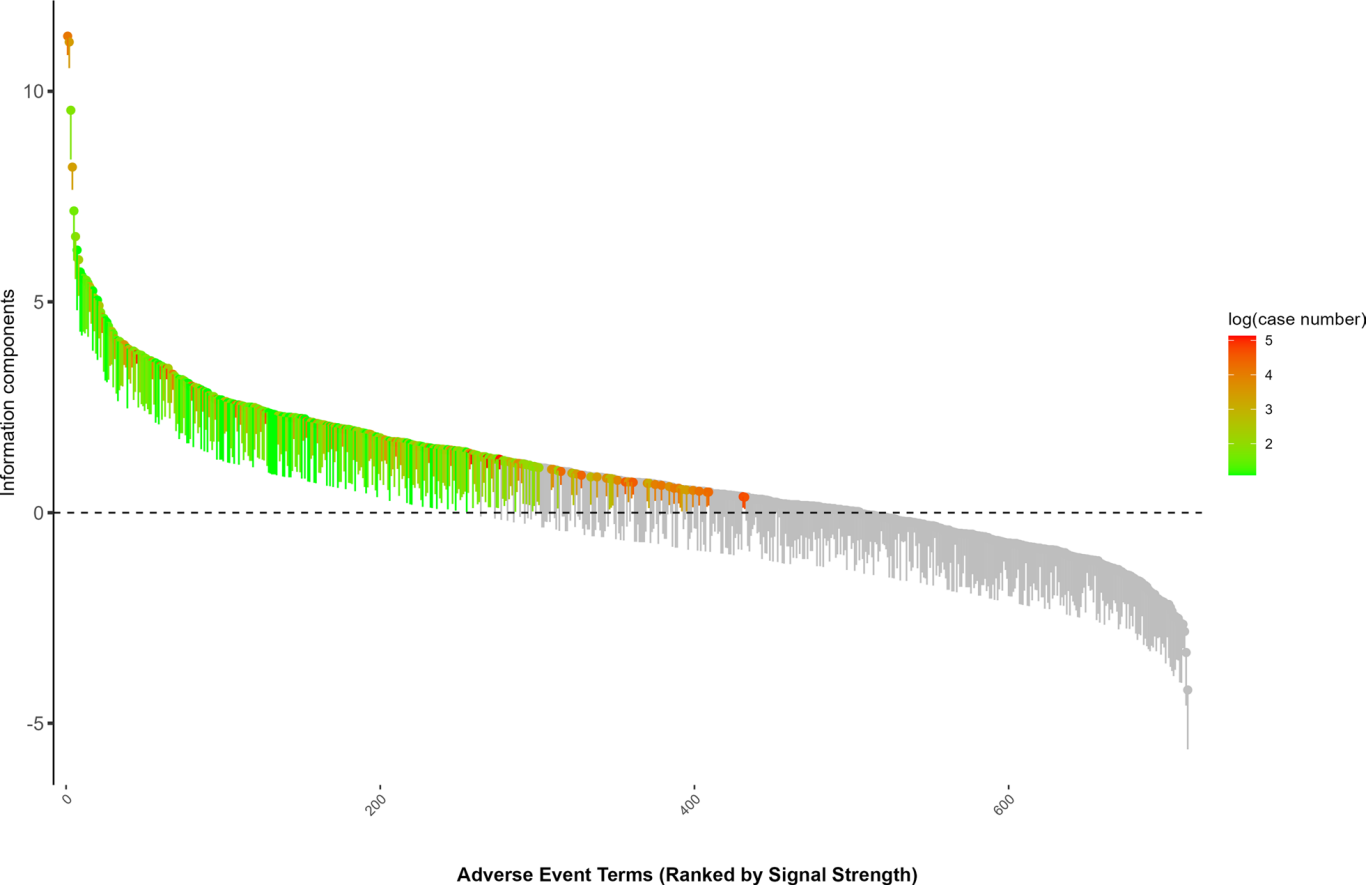
Waterfall plot of the top adverse event signals for imiglucerase ranked by signal strength. Each bar represents a unique adverse event term (Preferred Term), ranked by its Information Component (IC) value on the y-axis. The error bars indicate the 95% confidence interval for the IC estimate. The color gradient corresponds to the number of case reports for each term, and the horizontal dashed line at IC = 0 represents the threshold for signal detection

In analyzing the stability of the FAERS database, the adverse event report (AER) data from 2004 to 2023 exhibited some annual fluctuations and quarterly consistency. The data show that despite notable peaks in report numbers during certain years, such as 2009, 2015, and 2023, there is an overall stable trend. Particularly in recent years (2019–2023), the pattern of data collection and reporting has become more consistent, indicating a significant improvement in the reliability of the FAERS system (Fig. 3).

### Comparative signal analysis across four gaucher disease therapies

To investigate whether the strong adverse event signals detected for imiglucerase, such as bone infarction, were specific to the drug, a comparative analysis was performed against velaglucerase alfa, taliglucerase alfa, and eliglustat. The results, presented in Table 6, reveal distinct signal patterns among the therapies.

**Table 6:** Comparative signal analysis of key skeletal and indication-related adverse events for four Gaucher disease therapies

Preferred term (PT)	Imiglucerase (ERT)	Velaglucerase alfa (ERT)	Taliglucerase alfa (ERT)	Eliglustat (SRT)
	N (ROR, 95% CIN (ROR, 95% CI)	N (ROR, 95% CI)	N (ROR, 95% CI)	N (ROR,N (ROR, 95% CI)
Bone infarction	29 (316.62, 217.03–461.93)	6 (132.16, 58.94–296.35)	0 (No signal detected)	0 (No signal detected)
OsteonecOsteonecrosis	80 (11.21, 9.00–13.97)	26 (9.77, 6.65–14.37)	6 (5.58, 2.50–12.44)	5 (4.70, 1.95–11.30)
Bone pain	126 (10.78, 9.04–12.85)	52 (8.83, 6.72–11.61)	21 (8.99, 5.85–13.81)	19 (8.59, 5.47–13.50)
Gaucher disease	61 (6352.95, 4274.52–9441.98)	11 (1702.49, 884.83–3275.74)	3 (1271.79, 393.54–4109.96)	0 (No signal detected)

Data in cells are presented as N (ROR, 95% CI), where N is the number of case reports. A signal was considered detected if the lower bound of the 95% CI for the ROR was greater than 1. Abbreviations: PT, Preferred Term; ERT, Enzyme Replacement Therapy; SRT, Substrate Reduction Therapy; N, Number of reports; ROR, Reporting Odds Ratio; CI, Confidence Interval

^*^The PT"Gaucher disease"is a known indication-related reporting artifact and is included for comparative reference

The most striking finding is for Bone infarction. This PT generated a very strong signal for the two primary enzyme replacement therapies, imiglucerase (ROR 316.62) and velaglucerase alfa (ROR 132.16), but no signal was detected for taliglucerase alfa or the oral substrate reduction therapy, eliglustat. In contrast, signals for Osteonecrosis and Bone pain were present across all four drugs at a comparable magnitude, suggesting these are more general skeletal manifestations of Gaucher disease.

## Discussion

This study conducted an in-depth analysis of the FAERS database from the first quarter of 2004 to the fourth quarter of 2023, systematically evaluating adverse drug events (ADEs) associated with imiglucerase [19]. The findings revealed significant signals across various System Organ Classes (SOCs), offering supplementary insights to existing safety studies and providing new perspectives for clinical practice and pharmacovigilance [20].

### Background and comparison with existing research

Imiglucerase, an enzyme replacement therapy for Gaucher disease, has been widely used in clinical settings since its approval by the FDA in 1994 [21]. Gaucher disease is a lipid metabolism disorder caused by a deficiency of glucocerebrosidase, leading to symptoms such as splenomegaly, hepatomegaly, bone disease, and hematologic abnormalities [22]. Imiglucerase is administered via intravenous injection, with the dosage adjusted according to the patient’s weight and disease severity [23]. Although the efficacy of imiglucerase has been validated in numerous clinical trials, its safety profile in real-world settings remains a critical area for ongoing scrutiny [24, 25].

By mining data from the FAERS database, this study not only confirmed some of the existing safety information but also uncovered new potential risks [26, 27]. These findings align with those reported in the existing literature, further substantiating the safety concerns associated with the long-term use of imiglucerase [28, 29]. Notably, the study identified a significantly higher number of reports in several SOC categories, including general disorders and administration site conditions, injury, poisoning and procedural complications, infections and infestations, and nervous system disorders [1]. This suggests potential systemic risks associated with the drug, particularly under prolonged treatment. These findings underscore the importance of continued monitoring of imiglucerase’s safety in clinical practice, particularly in terms of its impact across different organ systems [25]. The study’s results highlight the need for clinicians to be vigilant about these potential risks, reinforcing the importance of individualized patient monitoring and the use of pharmacovigilance tools to ensure the safe and effective use of imiglucerase in treating Gaucher disease.

### Critical interpretation of signals: distinguishing reporting artifacts from confounding by indication

A rigorous interpretation of FAERS data requires distinguishing true safety signals from statistical artifacts and confounding factors [30]. Our analysis identified two primary examples of this challenge. The first was the top-ranked signal for the PT"Gaucher disease"itself (ROR = 6352.95). As addressed in the results, this is a clear reporting artifact. It represents a specific manifestation of a well-known methodological challenge where the indication for a drug can be misreported as an adverse event, a phenomenon conceptually rooted in what epidemiologists first described as protopathic bias [31]. Modern analyses of spontaneous reporting systems confirm that events like"drug ineffectiveness"or"condition aggravated"are often coded under the disease's PT, creating these large, spurious signals [32]. Recognizing and excluding such artifacts is a fundamental first step in valid pharmacovigilance.

The second, more complex, challenge was the exceptionally strong signal for Bone infarction (ROR 316.62) associated with imiglucerase. Initially, this could be interpreted as a significant drug-specific risk. However, our comparative analysis fundamentally reframes this finding. The signal for Bone infarction was similarly potent for velaglucerase alfa, another mainstream ERT, a finding consistent with literature suggesting comparable efficacy and safety profiles between these two agents [33]. In stark contrast, this signal was entirely absent for eliglustat (an SRT) and taliglucerase alfa, aligning with long-term clinical trial data for these drugs that did not report similar rates of adverse bone events [34].

This pattern strongly suggests that the intense signal for bone infarction observed in patients treated with imiglucerase and velaglucerase is not specific to the drugs themselves, but rather reflects the severity of the underlying Gaucher disease, which predisposes patients to higher risks for skeletal complications [35]. It is plausible that patients prescribed imiglucerase or velaglucerase represent a cohort with more severe disease phenotypes, who are inherently at a higher risk for skeletal complications like bone infarction. In contrast, patients on SRT (such as eliglustat) or the plant cell-expressed taliglucerase may represent a different, possibly less severe, patient subgroup, due to their different treatment mechanisms and patient selection criteria [36]. Therefore, the signal for bone infarction is most likely a manifestation of confounding by indication, rather than evidence of direct drug-induced toxicity, as suggested by recent literature [35]. This re-interpretation shifts the clinical implication from concerns over drug safety to the critical need for vigilant skeletal monitoring in high-risk Gaucher disease patient populations undergoing enzyme replacement therapy [37].

### Potential mechanisms for persistent disease manifestations during therapy

Furthermore, it is critical to explore the potential biological mechanisms that may underlie this observation, where even potent therapies like imiglucerase do not completely prevent severe outcomes like bone infarction in high-risk patients. A key potential mechanism involves an immune-mediated response to the therapy itself. It is well-documented that a subset of patients treated with imiglucerase develops anti-drug antibodies (ADAs) [38]. The presence of neutralizing ADAs can significantly impair the drug's efficacy by binding to the enzyme and blocking its activity [38]. This immune-mediated neutralization directly disrupts the intended metabolic pathway correction, leading to suboptimal substrate clearance and persistent Gaucher cell activity. This phenomenon could also explain the strong signals observed for enzyme level abnormalities, such as"Increased chitotriosidase”, which should be interpreted not as a drug side effect, but as a biomarker of inadequate therapeutic response [39]. Therefore, the"bone infarction"signal may represent a cohort of patients who not only have severe baseline disease but whose therapeutic response is being attenuated by an immune reaction. This shifts the clinical focus from a simple drug safety issue to the more complex challenge of monitoring for and managing treatment failure, potentially driven by patient-specific immunological factors [40].

### Clinical recommendations and practice

The findings of this study offer important guidance for clinicians. Firstly, while our analysis detected a signal for the System Organ Class (SOC)"Pregnancy, puerperium and perinatal conditions”, this finding must be interpreted with extreme caution as it highlights the profound limitations of FAERS for assessing materno-fetal risk. Drawing any conclusion about causality is impeded by several key factors inherent to the database: (1) the absence of a denominator, meaning the total number of pregnant women exposed to imiglucerase is unknown, making true risk calculation impossible; (2) significant reporting bias, where adverse outcomes like spontaneous abortion are more likely to be reported than healthy, full-term pregnancies, thus inflating disproportionality signals; and (3) confounding by disease, as Gaucher disease itself can impact pregnancy outcomes, an effect that cannot be disentangled from the drug's potential influence in FAERS data [41]. Close monitoring during pregnancy is recommended, as demonstrated in a case report where a woman treated with imiglucerase was carefully monitored throughout her pregnancy, leading to a healthy outcome despite the risks associated with the disease [42]. Additionally, for patients with hepatobiliary disorders, regular liver function monitoring is advised while using imiglucerase to detect and manage potential metabolic abnormalities promptly [43].

Secondly, and most critically, particular attention must be given to the management of bone health. While imiglucerase therapy significantly improves many systemic and hematological parameters, hallmark skeletal complications of Gaucher disease—such as bone pain and osteonecrosis—often persist and remain a primary concern for this patient population [44]. This aligns with our FAERS data, suggesting these events are manifestations of the underlying disease rather than direct adverse drug reactions. Therefore, our results underscore the critical need for vigilant, ongoing skeletal monitoring throughout long-term therapy. Proactive management is essential, as recent long-term studies confirm that the risk of fractures and avascular necrosis remains significant, even in patients who are otherwise stable on ERT [37, 45].

### Stability of FAERS database annual reports and data reliability

Our analysis indicates that despite some yearly fluctuations, the overall data trends within the FAERS database remain robust. This inherent stability is the cornerstone of modern pharmacovigilance, providing a reliable data foundation for long-term drug safety assessments. The successful application of FAERS for such longitudinal analyses is continuously affirmed by recent research, including safety studies on specific agents like cabozantinib [46]. Furthermore, the application of advanced methods, such as machine learning, to process and classify FAERS reports is vital for reducing data uncertainty and enhancing the accuracy of signal detection [47].

### Limitations of the study

This study has several limitations that should be acknowledged. The primary limitation arises from the use of the FAERS database, a spontaneous reporting system susceptible to significant underreporting and reporting bias [48].

Furthermore, a key limitation of our analysis is the inability to control for important confounding variables. For example, information on concomitant medications is often incomplete, making it difficult to attribute an adverse event solely to imiglucerase. Our study proactively addressed this challenge by incorporating a comparative analysis (section"Comparative signal analysis across four gaucher disease therapies"), which provided strong evidence that severe skeletal signals such as bone infarction are likely attributable to confounding by indication. While this methodological approach adds significant robustness to our interpretation, we acknowledge that without individual patient-level data on disease severity, this limitation cannot be entirely overcome.

Given these limitations, the disproportionality analysis used here can only generate signals and cannot establish causality. The findings should be interpreted as preliminary and hypothesis-generating. To overcome these limitations and validate our findings, future research is essential. We recommend prospective studies or analyses of comprehensive real-world evidence (RWE) databases (e.g., electronic health records or claims data) that allow for adjustment of confounders [49]. Additionally, designing case–control studies with carefully matched controls would provide a more rigorous method to assess the true risk associated with imiglucerase.

## Conclusion

This study, through an in-depth analysis of the FAERS database from 2004 to 2023, successfully identified significant adverse reaction signals for imiglucerase across multiple System Organ Classes. Our findings confirm the critical importance of continuous pharmacovigilance, especially in vulnerable populations such as pregnant women, long-term users, and patients with comorbid hepatobiliary or skeletal conditions.

Critically, our comparative analysis revealed that strong signals for severe skeletal events, like bone infarction, are most likely manifestations of confounding by indication reflecting underlying disease severity, rather than direct drug-induced toxicity. This finding underscores a crucial clinical reality: even with therapies that provide sustained clinical benefits over decades [50], significant challenges, such as the progression of skeletal disease, can persist in high-risk patients.

Therefore, future research should build upon these findings. Prospective studies are essential to delineate the complex metabolic mechanisms and true long-term effects of imiglucerase. This is particularly critical for optimizing treatment strategies in patient subgroups where such clinical challenges persist despite ongoing therapy [51]. Ultimately, this will help refine the clinical application of imiglucerase, ensuring its long-term safety is maximized alongside its proven efficacy.

## Supplementary Information

Additional file 1 Table S1: Drug name query terms for imiglucerase. This table lists all drug names, including brand names, generic names, and their variations, used to query and retrieve case reports associated with imiglucerase from the FAERS database.

Additional file 2 Table S2: Complete list of significant adverse event signals for imiglucerase. This table provides the full list of 101 Preferred Termsidentified as significant adverse event signals, organized by System Organ Class. It includes detailed disproportionality analysis statistics for each signal.

Additional file 3 File S1: R Script for Imiglucerase Analysis. This file contains the complete R code used for the data processing, signal detection, and primary analysis of imiglucerase adverse event reports presented in the study.

Additional file 4 File S2: R Script for Velaglucerase Alfa Comparative Analysis. This file contains the complete R code used for the comparative signal analysis of velaglucerase alfa.

Additional file 5 File S3: R Script for Taliglucerase Alfa Comparative Analysis. This file contains the complete R code used for the comparative signal analysis of taliglucerase alfa.

Additional file 6 File S4: R Script for Eliglustat Comparative Analysis. This file contains the complete R code used for the comparative signal analysis of eliglustat.

## Data Availability

The data utilized and analyzed during this study can be made available upon reasonable request to the corresponding author.
